# ADHD Remote Technology study of cardiometabolic risk factors and medication adherence (ART-CARMA): a multi-centre prospective cohort study protocol

**DOI:** 10.1186/s12888-022-04429-6

**Published:** 2022-12-20

**Authors:** Hayley Denyer, J Antoni Ramos-Quiroga, Amos Folarin, Carolina Ramos, Petra Nemeth, Andrea Bilbow, Euan Woodward, Susannah Whitwell, Ulrich Müller-Sedgwick, Henrik Larsson, Richard JB Dobson, Jonna Kuntsi

**Affiliations:** 1 Social, Genetic and Developmental Psychiatry Centre, Institute of Psychiatry, Psychology and Neuroscience, King’s College London, 16 De Crespigny Park, SE5 8AF London, UK; 2 Department of Psychiatry, Hospital Universitari Vall d’Hebron, Barcelona, Catalonia Spain; 3 Group of Psychiatry, Mental Health and Addictions, Vall d’Hebron Research Institute (VHIR), Barcelona, Catalonia Spain; 4 Biomedical Network Research Centre on Mental Health (CIBERSAM), Barcelona, Catalonia Spain; 5 Department of Psychiatry and Forensic Medicine, Universitat Autònoma de Barcelona, Barcelona, Catalonia Spain; 6 Department of Biostatistics and Health Informatics, Institute of Psychiatry, Psychology and Neuroscience, King’s College London, London, UK; 7 Institute of Health Informatics, University College London, London, UK; 8 NIHR Biomedical Research Centre at South London and Maudsley NHS Foundation Trust and King’s College London, London, UK; 9 Health Data Research UK London, University College London, London, UK; 10 NIHR Biomedical Research Centre at University College London Hospitals NHS Foundation Trust, London, UK; 11 Empatica Srl, Milan, Italy; 12 The National Attention Deficit Disorder Information and Support Service, ADDISS, Edgware, Middlesex UK; 13 European Association for the Study of Obesity – Ireland, Dublin, Ireland; 14 South London and Maudsley NHS Foundation Trust, London, UK; 15 Adult Neurodevelopmental Service, Health and Community Services, Government of Jersey, St Helier, Jersey; 16 Department of Psychiatry, University of Cambridge, Cambridge, UK; 17 Department of Medical Epidemiology and Biostatistics, Karolinska Institutet, Stockholm, Sweden; 18 School of Medical Sciences, Örebro University, Örebro, Sweden

**Keywords:** Attention deficit hyperactivity disorder, ADHD, Cardiovascular disease, Medication adherence, mHealth, Remote measurement technology, Remote monitoring, Digital phenotyping

## Abstract

**Background:**

Emerging evidence points at substantial comorbidity between adult attention deficit hyperactivity disorder (ADHD) and cardiometabolic diseases, but our understanding of the comorbidity and how to manage cardiometabolic disease in adults with ADHD is limited. The ADHD Remote Technology study of cardiometabolic risk factors and medication adherence (ART-CARMA) project uses remote measurement technology to obtain real-world data from daily life to assess the extent to which ADHD medication treatment and physical activity, individually and jointly, may influence cardiometabolic risks in adults with ADHD. Our second main aim is to obtain valuable real-world data on adherence to pharmacological treatment and its predictors and correlates during daily life from adults with ADHD.

**Methods:**

ART-CARMA is a multi-site prospective cohort study within the EU-funded collaboration ‘TIMESPAN’ (Management of chronic cardiometabolic disease and treatment discontinuity in adult ADHD patients) that will recruit 300 adults from adult ADHD waiting lists. The participants will be monitored remotely over a period of 12 months that starts from pre-treatment initiation. Passive monitoring, which involves the participants wearing a wrist-worn device (EmbracePlus) and downloading the RADAR-base Passive App and the Empatica Care App on their smartphone, provides ongoing data collection on a wide range of variables, such as physical activity, sleep, pulse rate (PR) and pulse rate variability (PRV), systolic peaks, electrodermal activity (EDA), oxygen saturation (SpO2), peripheral temperature, smartphone usage including social connectivity, and the environment (e.g. ambient noise, light levels, relative location). By combining data across these variables measured, processes such as physical activity, sleep, autonomic arousal, and indicators of cardiovascular health can be captured. Active remote monitoring involves the participant completing tasks using a smartphone app (such as completing clinical questionnaires or speech tasks), measuring their blood pressure and weight, or using a PC/laptop (cognitive tasks). The ART system is built on the RADAR-base mobile-health platform.

**Discussion:**

The long-term goal is to use these data to improve the management of cardiometabolic disease in adults with ADHD, and to improve ADHD medication treatment adherence and the personalisation of treatment.

## Background

Attention deficit hyperactivity disorder (ADHD) is a common psychiatric disorder, with a prevalence among adults of 2.5% [1]. The disorder is diagnosed based on impairing levels of inattentive, hyperactive and impulsive behaviours. Most adults with ADHD present with additional serious mental health problems that can include anxiety, depression, emotional instability, antisocial behaviour, substance use and autism spectrum disorder [2]. Emerging evidence further points at substantial comorbidity and shared genetics between adult ADHD and cardiometabolic diseases (obesity, type-2 diabetes mellitus and cardiovascular disease). However, detailed knowledge about the screening, diagnosis and clinical management of adults with ADHD and co-occurring cardiometabolic disease is lacking.

The prevalence of ADHD medication use for adults has increased markedly during the last two decades [3–5]. Randomised control trials (RCTs) on ADHD have shown short-term beneficial effects of ADHD medication on the core symptoms of ADHD [6–8]. However, concerns about the safety of ADHD medications for serious cardiometabolic outcomes have been raised due to reports of cardiovascular disease (CVD) events, including sudden cardiac death, myocardial infarction and stroke in individuals using ADHD medications [9–11]. ADHD medications may also elevate heart rate (HR) and blood pressure [12, 13]. Despite the evidence from the RCTs on short-term beneficial effects, there is a paucity of longer-term, real-world data on the impact of ADHD medication on a wider range of outcomes, as well as of such effects when combined with a non-pharmacological intervention, such as physical activity. The therapeutic effects of physical activity have been described extensively for several psychiatric and cardiometabolic conditions, including depression, sleep problems, obesity, type-2 diabetes mellitus and CVD [14–18]. There is also emerging evidence for positive effects of physical activity on ADHD [19–24], but long-term real-world data are needed, in particular among adults.

Possible reasons for treatment discontinuity in adults with ADHD include lack of treatment response, adverse side effects and poor adherence behaviours (such as forgetting to take the medication). Although sub-optimal response is one of the most common reasons reported for ADHD medication discontinuation, few studies report on adherence and discontinuation results [25]. A systematic review found that of 91 observational studies only eleven studies reported adherence or discontinuation results in adults with ADHD [25]. Side effects reported to ADHD medication include changes to sleep behaviours (such as reduced total sleep time and delayed onset of sleep [26]), reduction in appetite and weight [27, 28]) and hypertension [10, 13, 28]. RCTs or observational studies that rely exclusively on parent/caregiver surveys often do not provide longer-term information about how side effects may influence ADHD medication treatment discontinuity [10, 29]. To improve our understanding of the reasons for treatment discontinuity in adults with ADHD, moment-by-moment quantification of real-world data from the individuals’ daily lives including both active (e.g., completing questionnaires) and passive (e.g., wearable device) monitoring is needed.

Remote measurement technology (RMT) offers new opportunities for collecting detailed, frequent, long-term, real-world data on clinical symptoms, functional and cognitive impairments, physiological measures, health behaviours (such as exercise and sleep) and measures of the environment on large sample sizes. The ADHD Remote Technology (‘ART’) assessment and monitoring system for adults with ADHD, developed by Kuntsi, Dobson and colleagues [30, 31] utilises this technology and consists of both active and passive monitoring. Active remote monitoring involves the participant completing tasks using a smartphone app (such as completing clinical questionnaires or speech tasks) or using a PC/laptop (cognitive tasks). Passive remote monitoring consists of ongoing data collection using smartphone sensors and a wearable device without active input from the participant; data can be collected on a wide range of variables, such as physical activity, sleep, pulse rate (PR) and pulse rate variability (PRV), systolic peaks, electrodermal activity, oxygen saturation (SpO2), peripheral temperature, smartphone usage including social connectivity, and the environment (e.g. ambient noise, light levels, relative location). The ART system is underpinned by the RADAR-base open-source mobile-health platform [32, 33].

This protocol describes the ADHD Remote Technology study of cardiometabolic risk factors and medication adherence (‘ART-CARMA’), which uses the ART system. ART-CARMA is a clinical study within the EU-funded collaboration ‘TIMESPAN’ (Management of chronic cardiometabolic disease and treatment discontinuity in adult ADHD patients), which unites a multidisciplinary team of 17 partners from academia, small and medium-sized enterprises (SMEs), patients and care providers. The main objective of TIMESPAN is to advance the management of adult ADHD and co-occurring cardiometabolic disease by improving the identification and treatment of individuals with these comorbidities. The wearable device included in ART-CARMA is the EmbracePlus developed by the SME partner Empatica [34]. ART-CARMA will use RMT in adults with ADHD to carry out unobtrusive, real-time data collection over a period of 12 months. By recruiting 300 adults from adult ADHD clinic waiting lists and monitoring them remotely, we will obtain objectively measured data relevant to cardiometabolic risk profiles from the patient’s daily life, both on and off pharmacotherapy. By targeting the informative period from pre-treatment initiation through to treatment initiation, titration and the subsequent period, up to 12 months in total, we will obtain real-time data on multiple parameters, including side effects, that aims to inform future personalisation of treatment.

### Patient involvement in the ART-CARMA study protocol

The ADHD Remote Technology (ART) system benefits from procedures developed in the RADAR-MDD study protocol [35]; a RMT study that recruited over 600 individuals with major depression disorder (MDD), over a 24-month remote monitoring period. RADAR-MDD underwent extensive development in collaboration with service-users to promote the design of acceptable and feasible RMT tools [36]. In addition, study participants from our initial ART pilot study provided feedback on the acceptability of measures and procedures piloted in that study [31]. In ART-pilot, 95% of the participants agreed that there is value in gathering data through RMT, with some participants explaining passive monitoring provides a unique opportunity to collect objective data. All participants with and without ADHD (100%) agreed that the study measures blended into their daily life, and 82% of participants were positive about using the system for at least one year. We have incorporated improvements to the ART-CARMA study design that were suggested by the participants in ART-pilot, such as longer intervals between the administration of the cognitive tasks during the remote monitoring period. We also ran a service-user focus group that further informed this study design regarding the frequency of questionnaire administration and the acceptability of additional measures included in ART-CARMA that were not included in ART-pilot. We have incorporated suggestions from TIMESPAN partners Andrea Bilbow and Euan Woodward. Andrea Bilbow, a patient advocate for people with ADHD, founder and Chief Executive of Attention Deficit Disorder Information Services (ADDISS), has provided input to the ADHD remote measure development via the wider ART research programme (on which she is an external collaborator). She has provided further input into the ART-CARMA study protocol specifically during the initial planning stage. Mr Euan Woodward, executive director of the European Association for the Study of Obesity (EASO) has been involved in the project outline.

### Study objectives

The first main aim (objectives 1–3 below) is to obtain real-world data from the patient’s daily life on the extent to which ADHD medication treatment and physical activity, individually and jointly, may influence cardiometabolic risks in adults with ADHD. This will provide new insights into disease patterns and help improve the safety and effectiveness of pharmacological (i.e., ADHD medication treatment) and non-pharmacological (i.e., physical activity) interventions for patients with ADHD and co-occurring cardiometabolic disease. The second main aim (objectives 4–6 below) is to obtain in vivo, real-world data from the patient’s daily life on adherence to pharmacological treatment and its predictors and correlates, over a remote monitoring period of 12 months that starts from pre-treatment initiation. The long-term goal is to use these data to improve the management of cardiometabolic disease in adults with ADHD, and to improve ADHD medication treatment adherence and the personalisation of treatment.

ART-CARMA has six specific objectives:

- Objective 1: Use RMT to identify real-world consequences of ADHD medication treatment on the following cardiometabolic risk (or protective) factors in adults with ADHD: changes in HR, blood pressure, weight, smoking, alcohol use, diet, sleep and physiological stress response.
- Objective 2: Use RMT to measure the potential contribution of physical activity to a reduction in these risk factors, independently and in combination with ADHD medication treatment.
- Objective 3: Use machine learning, applied to the full set of multimodal remote monitoring data from the 12-month period, to build prediction models of the cascade of effects that follow from the initiation of ADHD medication treatment. This allows us to go beyond objectives 1 and 2, to obtain insight into potential underlying mechanisms that are needed for developing algorithms for personalised treatment. The prediction models allow us to assess complex, temporal relationships between multiple measures.
- Objective 4: Use RMT to explore ADHD medication use patterns in adults with ADHD in the first year of starting ADHD medication treatment (including treatment initiation, titration and the subsequent period) and to quantify the extent of treatment adherence, non-adherence and treatment gaps.
- Objective 5: Use RMT to identify potential reasons for non-adherence, with a focus on adverse side effects (e.g. daytime sleepiness and changes in electrodermal activity indexed arousal/alertness, changes in heart rate or blood pressure, changes in appetite/weight, increased anxiety/depressive symptoms or irritability, not feeling themselves/feeling their personality changes when on medication, tics, dizziness, sweating, headaches), lack of treatment response (limited or no effect on ADHD symptoms and impairments) and poor adherence behaviours (e.g. forgetting to take or difficulties taking ADHD medication). We capture these time series data across the periods of pre-treatment, treatment initiation, titration and subsequent monitoring.
- Objective 6: Identify psychiatric comorbidities that predict treatment non-adherence or increase adverse effects. We will measure symptoms of autism spectrum disorder, antisocial behaviour, anxiety, depression and substance use.

## Methods

### Study design

ART-CARMA is a clinical study being undertaken as part of a larger body of work by the Management of chronic cardiometabolic disease and treatment discontinuity in adult ADHD patients (TIMESPAN [37]) research consortium. Below is a summary of the methods used in ART-CARMA.

ART-CARMA is a prospective observational non-randomised, non-interventional study, using wearable technology and smartphone sensors, representing no change to the usual care or treatments of participants due to participation. There is no control comparison group, or randomised group allocation.

### Study population

Power calculations (GPower 3.1) for a regression analysis with five predictors indicate that a sample size of 280 has 80% power (alpha 0.01) to detect a small effect size (f2) of 0.07. Published Monte-Carlo simulations that aimed to determine the necessary sample size to achieve stable estimates for correlations suggest that, in typical scenarios, a sample size of approx. 250 is required for stable estimates [38]. These considerations, together with the evidence that has accumulated from the RADAR-MDD remote monitoring study regarding potential challenges with RMT protocols (e.g., attrition, missing data) ([39]), have led to the proposed sample size of 300. The eligibility criteria for inclusion in this study are summarised in Table 1.

**Table 1:** Eligibility criteria for participation in ART-CARMA

ADHD group inclusion criteria	Diagnosis of DSM-5 ADHD
	Aged 18–60
	Able to give informed consent for participation
	Fluent in English
	Willing and able to complete self-reported assessments via smartphone
	Willing to use either their own Android phone or a study Android phone as their only smartphone during the data collection period
	Willing to wear the wearable device (EmbracePlus) during the data collection period
	Not on ADHD medication at the time of recruitment
ADHD group exclusion criteria	Psychosis, currently experiencing a major depressive episode, mania, drug dependence over the last six months or a major neurological disorder
	Recent contact with psychiatric acute care (admission, crisis team or liaison team (A&E)) in the last six months
	Any other major medical disease which might impact upon the patient’s ability to participate in normal daily activities (e.g., due to hospitalisations)
	Does not start ADHD medication following ADHD diagnosis (either due to personal choice or psychiatrist deciding not to prescribe ADHD medication)
	Pregnancy
	IQ < 70
Partner, family member or close friend group inclusion criteria	A parent, partner or a close friend, as chosen by the participant with ADHD
	Aged 18 or over
	Willing and able to complete web-based questionnaires regarding the participant with ADHD

*DSM-5* The Diagnostic and Statistical Manual of Mental Disorders, Fifth Edition, *IQ* intelligent quotient

### Study procedures

**Fig. 1:**
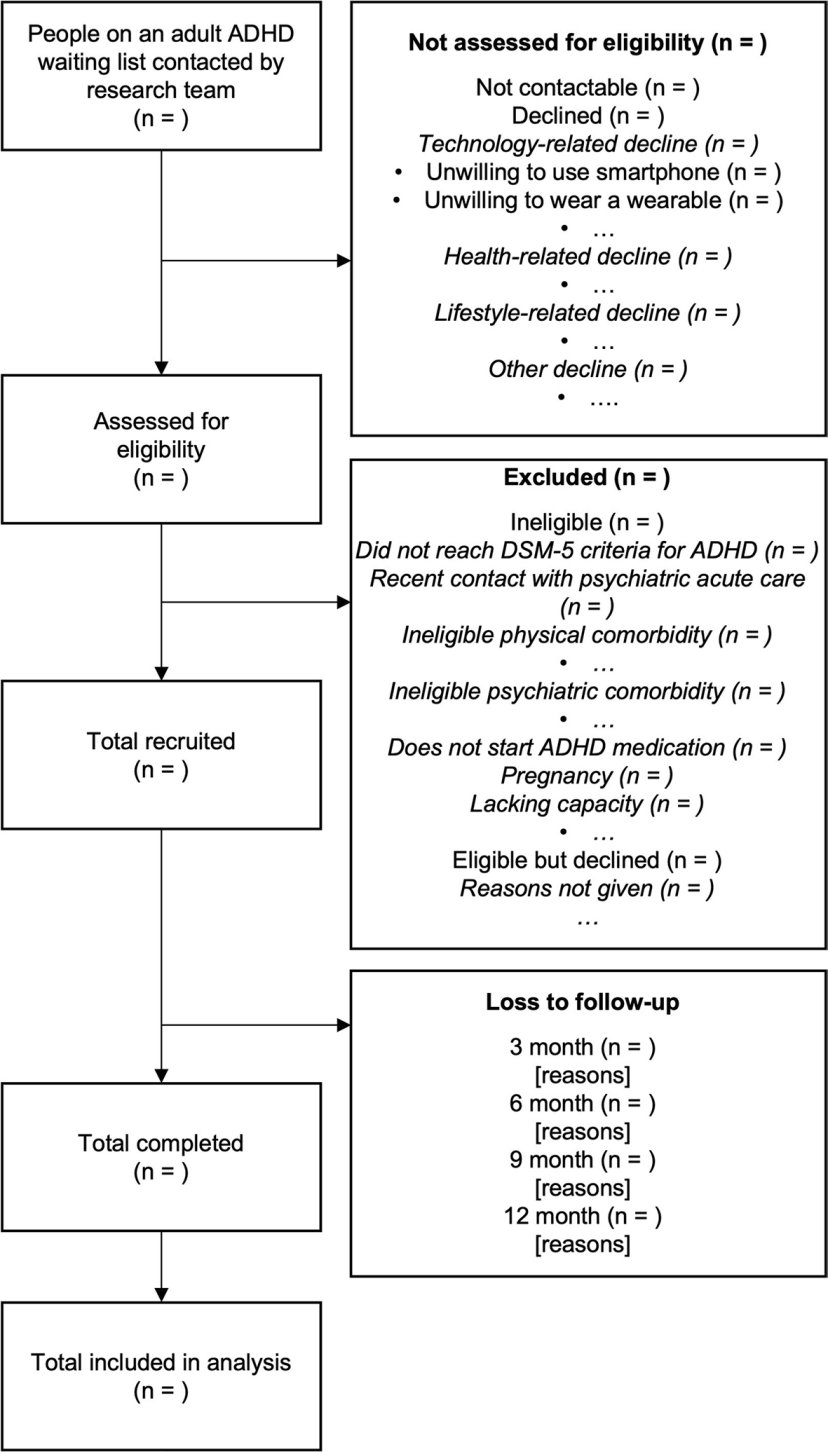
Participant flowchart

Participants will be recruited over twenty-four months across two data collection sites of ART-CARMA at King’s College London (UK) and Vall d’Hebron Research Institute (Spain). In total, across the two sites, we will recruit 300 adults from adult ADHD clinic waiting lists, which enables an initial off-medication remote monitoring period and subsequent continuous remote monitoring following initiation of ADHD medication treatment. The schedule of events is available in Table 2. The flow of participants through the study, including reasons for not participating in the study, will be standardised across sites and documented in a flowchart (Fig. 1).

**Table 2:** Schedule of events for ART-CARMA

Month	0		1	2	3	4	5	6	7	…	11	12
Assessment	1	2	n/a	n/a	n/a	n/a	n/a	n/a	n/a	n/a	n/a	n/a
Baseline assessments												
Study explanation (WR)	X											
Informed consent (WR)	X											
Introductory training (WR)		X										
ADHD diagnostic interview DIVA-5 (WR)	X											
Psychiatric comorbidities MINI (WR)	X											
IQ and working memory WASI-II and WAIS-IV (WR)	X											
Socio-demographics, medical history, service use, experience with technology (R/WR)	X											
Autism Spectrum Disorder AQ-10 (R/WR)	X											
Cognitive tasks (WR)		X						X				X
Physical measures of weight and waist circumference (WR)		X										
Blood pressure (WR)		X										
DNA saliva sample		X										
Microbiota sample		X										X
Remote data collection												
Wearable sensors			Continuous (months 1-12)									
Smartphone sensors (pRMT)			Continuous (months 1-12)									
Medication use (aRMT)			Continuous (months 1-12)									
Medication side effects CADDRA Patient ADHD Medication Form (aRMT)			XX XX	XX XX	XX XX	XX XX	XX XX	XX XX	XX XX	XX XX	XX XX	XX XX
ADHD BAARS-IV – self-report (aRMT)		X	X	X	X	X	X	X	X	X	X	X
Anxiety GAD-7 (aRMT)		X	X	X	X	X	X	X	X	X	X	X
Depression PHQ-8 (aRMT)		X	X	X	X	X	X	X	X	X	X	X
Aggression RPQ-A (aRMT)		X	X	X	X	X	X	X	X	X	X	X
Irritability ARI – self-report (aRMT)		X	X	X	X	X	X	X	X	X	X	X
Weight and waist circumference (aRMT)		X	X	X	X	X	X	X	X	X	X	X
Blood pressure (aRMT)		X	X	X	X	X	X	X	X	X	X	X
Smoking Test Fagerstrom (aRMT)		X	X	X	X	X	X	X	X	X	X	X
Alcohol use AUDIT (aRMT)		X	X	X	X	X	X	X	X	X	X	X
Diet 14-item Mediterranean diet adherence questionnaire (aRMT)		X	X	X	X	X	X	X	X	X	X	X
Speech sample task (aRMT)		X	X	X	X	X	X	X	X	X	X	X
Major life events LTE (aRMT)		X						X				X
ADHD BAARS-IV – other report (R)		X	X	X	X	X	X	X	X	X	X	X
Irritability (ARI – informant report) (R)		X	X	X	X	X	X	X	X	X	X	X

*WR* With researcher either in-person or virtually, *R* REDCap web-based platform, *pRMT* Passive App, *aRMT* Active App. X delivered once per month. XX delivered twice per month (every 2-weeks). XXXX delivered four times per month (every week)

Recruitment procedures will vary slightly across the two sites. Adults will be recruited who are on the waiting list and are not yet receiving ADHD medication. In the UK/British Isles, the clinical team will contact the individuals by their preferred method of contact (post, email, or telephone) and will provide them with information about the study. If the patient is interested in taking part, the first baseline assessment with the research team will be scheduled. The participant information sheet and informed consent form will be sent to patients a minimum of 24 h before the baseline assessment. In Spain, individuals will be provided with information about the study on the day of their ADHD assessment. If the patient is interested in taking part, the baseline assessments will be completed on the same day. Written informed consent will be obtained.

The baseline assessments will happen either in-person or virtually using Microsoft Teams depending on the site they have been recruited from. In the UK/British Isles, participants will be paid £30 at the point of enrolling in the study, and a further £5 for every completed outcome measure (paid at months 6 and 12). Participants will be paid an additional £30 at the end of the study period (month 12). In Spain, participants will not receive any financial compensation for their participation.

The baseline assessments will take approximately four hours to complete. In Spain, the two in-person baseline assessment sessions will be completed in one day. In the UK/British Isles, the virtual baseline assessment sessions will be completed across two days, with approximately a week in between each session. Both the in-person and virtual baseline sessions will consist of (1) the Diagnostic Interview for ADHD in Adults (DIVA [40]) to confirm an ADHD diagnosis, (2) a brief IQ and short-term working memory test [41, 42], (3) Mini International Neuropsychiatric Interview (MINI [43]) to establish psychiatric comorbidities, (4) two baseline questionnaires: a demographic About You questionnaire and the Autism Spectrum Quotient questionnaire (AQ-10 [44]), (5) two cognitive tasks of the Combined cued continuous performance test and Go/NoGo (CPT/GNG) task and the Fast task (while researcher present), (6) completion of questionnaires and physical measurements of weight, waist circumference and blood pressure, (7) training session on the smartphone, wearable device and blood pressure machine, and (8) guidance on how to collect DNA saliva sample and microbiota sample (optional). Participants will be able to take breaks during the session if required.

During the baseline session, the participants with ADHD will also be asked to identify a partner, a family member or a close friend who could complete informant-report versions of the active monitoring questionnaires on ADHD symptoms and impairment, and irritability, using the Research Electronic Data Capture (REDCap), a web-based platform. The research team will contact the partner, family member or close friend (via email or telephone) and invite him/her to complete the questionnaires every four weeks.

### Remote data collection

**Fig. 2:**
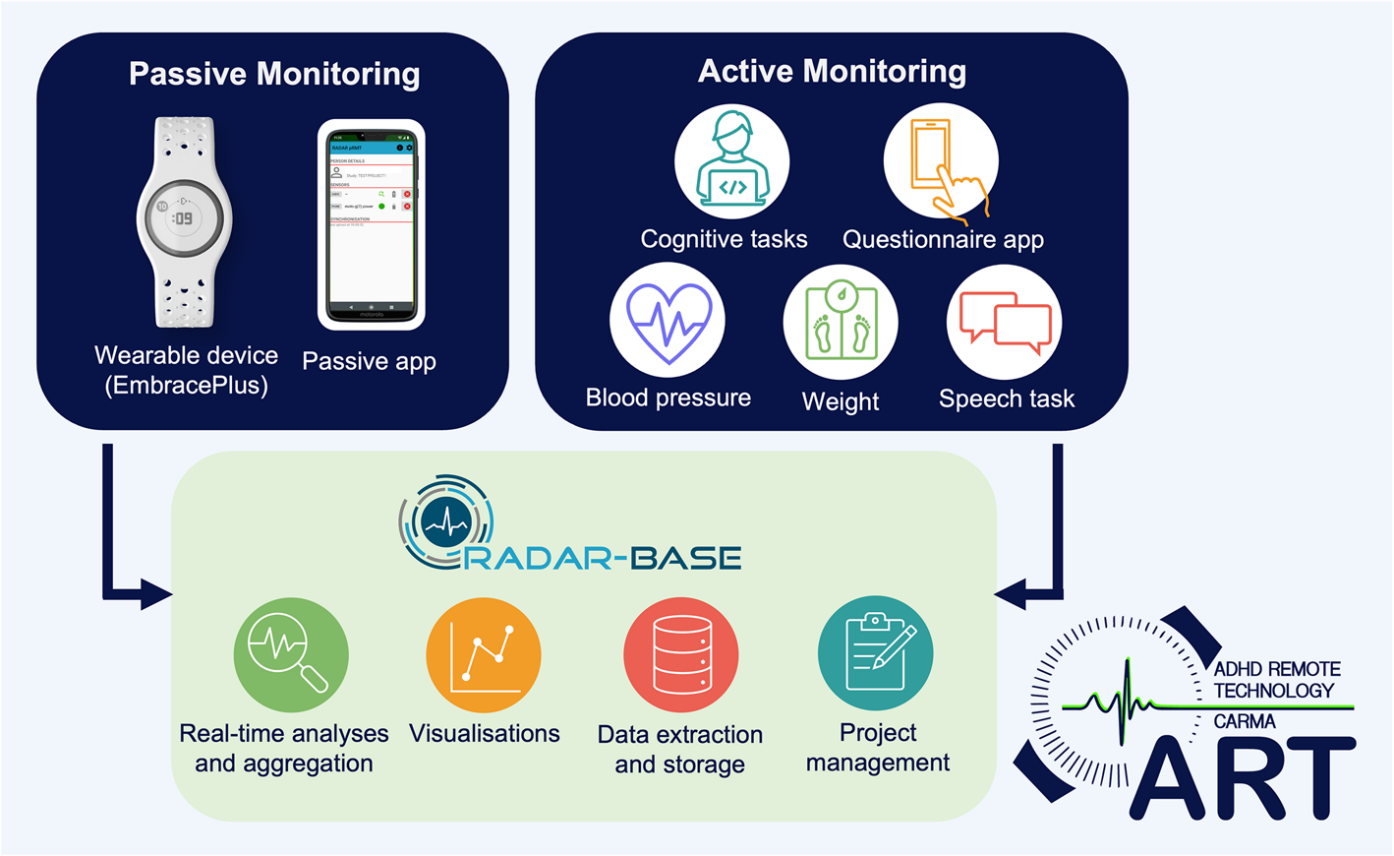
ART-CARMA active and passive monitoring

The﻿ r﻿emote monitoring starts immediately following the research baseline assessment and will last for 12 months. The remote monitoring consists of both active and passive components, following procedures established for the wider ART research programme, and is linked to the RADAR-base mobile-health platform [32]. Below is a summary of the methods of remote data collection used in the ART-CARMA study (Fig. 2).

#### Active App

Participants will be asked to download the RADAR-base Active App that requests the participant to complete questionnaires at specific intervals. Every day participants will be asked to complete a short questionnaire for medication use on whether the participant took their medication that day, with follow-on questions on type and dose of medication or why medication was not taken (e.g., experienced side effects, forgot to take their medication, or chose not to take their ADHD medication that day). Every week participants will be asked to report their medication use side effects using the CADDRA Patient ADHD Medication form [45]. This form contains four items on changes experienced since starting medication and 28 items on side effects. Every four weeks, participants will be asked to complete a longer set of questionnaires: ADHD symptoms and impairments will be measured using the Barkley Adult ADHD Rating scale on current symptoms (BAARS-IV) [46] and Barkley ADHD functional impairment scale [46]. This includes the 18 diagnostic ADHD symptoms and 10 items on functional impairment; Depression will be measured using the 8-item Personal Health Questionnaire Depression Scale; PHQ-8 [47], which is a widely used measure of current depression in the general population; Anxiety will be measured using the 7-item Generalized Anxiety Disorder questionnaire; GAD-7 [48], which provides a dimensional score of anxiety symptoms; Aggression will be measured using the 23-item Reactive-Proactive Aggression Questionnaire for Adults; RPQ-A [49]; Irritability will be measured using the self-reported 7-item Affective Reactivity Index questionnaire; ARI-s [50]; Smoking behaviours will be measured the 6-item Test Fagerstrom questionnaire [51]; Alcohol use will be measured using the Alcohol Use Disorders Identification Test; AUDIT [52]; Diet will be measured using the 14-item Mediterranean diet adherence questionnaire [53]; Blood pressure and weight measurements will also be taken. Participants will be provided with an Omron M2 Basic blood pressure monitoring machine, weighing scales and a tape measure; Major life events will be measured every 6 months using the 13-item List of Threatening Experiences; LTE [54]. The participant will also be asked to complete a short speech task monthly, on the Active App.

Participants will also self-administer the two cognitive tasks of CPT/GNG and Fast task twice during the 12 months (months 6 and 12) on their home PC or laptop, without the researcher present. In addition, ratings on ADHD symptoms are obtained monthly also from a partner, a family member or a close friend of the individual with ADHD using web-based Redcap.

#### Passive App

Participants will be asked to download the RADAR-base passive remote monitoring app, configured for ART requirements [32], which will run in the background, requiring no further input from participants. The Passive App collects data on ambient noise, ambient light, phone usage information (e.g., which apps were used and how long, when the phone was unlocked), passive audio, Global Positioning System (GPS) location, Bluetooth connectivity, battery life, gyroscope, steps and acceleration. Features of the passive audio, rather than raw audio itself, are extracted from the audio on the phone for transmission. GPS location data will be randomised; that is, providing relative location data, not absolute coordinates. This prevents identification of an individual’s home address or precise geographical location.

#### Wearable sensors

The wearable device in ART-CARMA is the Empatica EmbracePlus [34]. Participants will be asked to wear the device for the duration of the study (12 months), which will provide ongoing data collection of PR, PRV, systolic peaks, electrodermal activity, oxygen saturation (SpO2), peripheral temperature, and acceleration. By combining data across these variables measured, processes such as physical activity, sleep, autonomic arousal, and indicators of cardiovascular health can be captured.

#### Empatica Care App

The Care App collects data from the EmbracePlus and sends it to the Empatica Cloud, where it is stored. The data collected by the EmbracePlus is transferred to the Care App via Bluetooth®. The Care App then transmits the data to the Empatica Cloud via WiFi. To access and use the Care App, the participant has to scan the QR code or type in the participant ID/password provided by the research team. The login credentials generated by the research team via the Care Portal are unique and de-identified.

#### Compatible Android smartphone

Both the Passive App and the EmbracePlus require compatible smartphones to be used in ART-CARMA. The Passive App requires an Android smartphone and the EmbracePlus requires Bluetooth® 5.0. For participants who do not own a smartphone, we will provide a compatible Android smartphone for them. For participants who are using a non-Android or a non-compatible Android smartphone, we will not recruit them unless they agree to switch to a compatible Android smartphone as their only phone for the duration of the study. We will provide a compatible Android smartphone for them.

### Outcome measures

The outcome measures are (i) the cardiometabolic risk factors (HR, blood pressure, weight, smoking, alcohol use, diet, sleep, physiological stress response) and (ii) daily medication use (adherence), adverse side effects and other reasons for non-adherence.

#### Process evaluation

To ensure a system which meets participants and researcher needs, there will be continued monitoring of the ART system throughout the data collection period, including participant adherence to wearable devices and smartphone, and completion of measures. At the study endpoint, we will offer post-participation qualitative interviews to participants. Interviews will be offered to all participants who completed to the study end-point and to those who dropped out during their 12-month study period.

#### Follow-up procedure and data monitoring

Participants will be contacted by telephone after one month of the baseline assessment to address any concerns or questions. Brief follow-up phone-calls will then be offered every three months to maintain engagement with participants throughout the course of the study and to remind participants of any upcoming assessments. Participants will be encouraged to live their lives as normal, responding to the Active App notifications when required. The research team will be able to review incoming data using the RADAR-base management portal and Empatica Research Portal. The research team may contact participants if there is a loss of data from a device. All contact with participants will be recorded as evidence of feasibility and acceptability of outcomes.

### Study debrief

At the end of the follow-up period, participants will receive a ‘debrief’ session, which will serve to collect end-point acceptability and usability outcomes, and to retrieve study materials (devices, chargers etc.). This will be done either over the phone or on Microsoft Teams with a member of the research team and will be audio-recorded using Microsoft Teams. Participants will also have an opportunity to view and discuss their own data.

### Adverse events and study withdrawal

There may be several reasons for withdrawal from the study:

1. Participant choosing to no longer participate: participants will be informed both in writing and verbally that participation is voluntary, that non-participation will not influence their medical care, and that they are free to withdraw from the study at any point without providing reasons.
2. The research team may withdraw the participant in the event of inter-current illness, adverse event (AE), protocol violation, administrative or other reasons.

In the case of participant self-withdrawal, all attempts will be made to follow up with the participant to establish cause of withdrawal, and to collect qualitative data regarding experience of participation. All data, including those from withdrawn participants, will be included in the final analysis. If a participant withdraws from the study prematurely, we will consider replacing the participant if resources allow and if recruitment is ongoing for the study.

### Statistical and analysis plan

Descriptive statistics for demographics, attrition rate and number of participants using the remote assessment measurements without loss or damage and providing adequate data for the duration of the study period will be estimated. Using a survival model approach [55] and clustering analyses, we will investigate whether any demographics and/or other numerical information obtained during baseline of the study might serve as a predictor for subject drop out/percentage of adequate data. Using mixed-effect models with participants as a random factor, we will estimate whether relationships between the amount of usable data during the weeks prior to the outcome assessment and obtained scale’s measurements exist.

Regression and machine learning approaches will be used to investigate the changes in cardiometabolic factors measured by RMT (listed in Objective 1) following initiation of ADHD treatment. Data obtained from the accelerometer will be used to label days/part of the days with different gradation levels of activity levels and the duration of such activities on a per day basis. For Objective 2, Activity and derived features will be used to statistically test the effect of average activity level on cardiometabolic factors defined in Objective 1 with/without treatment. As well as typical statistical modelling approaches, this analysis will utilise both conventional machine learning methods including support vector machines and random forest in combination with feature selection and fusion approaches, as well as more contemporary deep learning methods (Objective 3).

Actigraphy read-outs will also be used to derive sleep characteristics using Empatica’s proprietary, 2-step algorithm that automatically identifies sleep periods and within the determined sleep period classifies epochs as sleep or wake with a 1-minute granularity. Sleep data will be further analysed in relation to Active App data and outcome scales. Utilizing EmbracePlus’ sensor suite, sleep analyses can further be refined by looking at sleep and wake timing, wake bouts, sleep efficiency and fragmentation, activity level(s) over the day during wake (based on 3-axis accelerometers), several features of EDA during wake (gated with motion and temperature) and sleep (including sleep storm patterns). Empatica’s team is also able to post-process EmbracePlus’ sleep or rest data to obtain estimates of respiration and PR, controlling for several factors (movement artefact and compliance) in its processing [56–59]. Raw, regularly sampled blood volume pulse (BVP) data will be used to estimate PR and PRV metrics and respiratory dynamics that result from the interplay of the sympathetic and parasympathetic nervous system. The extraction of features from the homogeneous sample of remotely monitored biosignals will be done according to well-established and openly documented methods (e.g., Task Force of the European Society of Cardiology and the North American Society of Pacing and Electrophysiology). Artefacts will be detected, and the signal noise will be appropriately filtered when it is possible. Spectral and time-domain features extracted from continuous biosensor data will be aggregated across a period of time using statistical characteristics, aligned and collated with features extracted from smartphones, assessments and Redcap, for statistical analysis.

Medication use clustering will be analysed for details around behaviour and pattern of use in Objective 4 and extended in Objective 5 to look at specific predictors of use. Time-variant clustering will be used to define subgroups of medication use classes across time, including the probability of transitioning between classes using mixture latent Markov models.

Machine learning will be implemented in order to build individualised prediction models for treatment response, symptom classification and stratification, and medication adherence (Objective 6). Based on previous work, different prediction models including Neural Processes model personalization and meta-learning techniques, developed for few-shot learning to individualize models, will be used [60].

### Data protection

We have ensured the highest standards of data security are in place. All electronic data collected will be streamed to Amazon Elastic Kubernetes System (EKS) server where the RADAR-base platform will be deployed for data collection. Amazon EKS servers will be located in London (UK), adhering to General Data Protection Rules (GDPR). Access to RADAR-base stack on Amazon EKS is restricted to system administrators. Data will be placed within the secure network of King’s College and Rivest–Shamir–Adleman (RSA) asymmetric public key encryption will be applied to access. All the data will be stored anonymised and highly restricted access will be given to specific authorised researchers. Industry grade security will be used to secure data access. Authorised access to secure Storage Server using RSA encryption over Secure Shell (SSH-RSA), sFTP access, Access Control Lists (ACLs) and user-groups will be used to control object-level permissions in the storage for each user. EmbracePlus data collected by Empatica will be pseudonymised (dummy information, such dummy name and email address will be entered into the Empatica accounts). Information sheet and consent forms provide details about how participants personal data is handled. Participants will also have the opportunity before giving sign consent to ask the research worker any questions regarding this. All the personal information will be handled according to GDPR and this will be explained in detail to the participants.

Empatica Cloud is the cloud infrastructure where the pseudonymised data recorded by the EmbracePlus is collected and made accessible via web interfaces and data repositories. Data is securely stored and encrypted within an Amazon Web Services (AWS) S3 bucket located in the United States (N. Virginia), and then pulled to secure sFTP storage placed in KCL (with the same authorisation and access controls discussed above). Data is encrypted at rest and in transit. Access to this data is granted by a set of unique, secret keys provided by Empatica. The Empatica Portal is GDPR compliant and a Data Processing Agreement has already been established between the research parties and the participant information sheet and consent form includes information about the wearable sensor data.

To maintain participant anonymity, their data will be pseudonymised. Their personal information will be stored separately in REDCap from information collected from the apps and wearable. Participant’s name will be replaced with a code, which will be randomly generated and a combination of a human readable ID (usually a number), project name, and site location, and the information collected from the apps and wearable will only be associated with this code. Information from the wearable and smartphone apps will be encrypted (scrambled) so that only the research team can see it. Transfer of data between different components is secured using industry standards. Data extracted from the platform are also secure and private; only people who have rights can access it. Pseudonymised data will be securely stored on servers managed by the Institute of Psychiatry, Psychology and Neuroscience, KCL, and also on 3rd party servers, which can provide additional security and backup. These data will not be linked back to participants personal information. All details of how data is pseudonymised will be included in the Information Sheets and Informed Consent Forms.

## Discussion

Using RMT, ART-CARMA will obtain real-world data on ADHD medication adherence and its predictors, and on the extent to which ADHD medication treatment and physical activity may influence cardiometabolic risks in adults with ADHD. Detailed digital phenotyping has not been captured in previous large real-world studies that have relied on health record databases.

ART-CARMA benefits from the recent development of the ADHD Remote Technology (ART) system [30, 31], the RADAR-base platform [33] and the new EmbracePlus wearable device from our SME partner Empatica [34]. The multi-parametric measurements over 12 months, starting from the pre-treatment initiation period, would not be possible using other methods. The phenotyping covers detailed physiological measures, health behaviours (such as physical activity and sleep), self-reported clinical symptoms, medication adherence and medication side effects, cognitive performance, speech samples and passive monitoring data on the environment and digital behaviours.

The long-term aim for ART-CARMA is to improve the management of cardiometabolic disease in adults with ADHD, and to improve ADHD medication treatment adherence and the personalisation of treatment. RMT uniquely has the potential, in the future, to offer the clinician easy and time-saving access to detailed data on symptoms and impairments during the titration process and subsequent long-term monitoring of treatment effects, which could help improve patient outcomes in a cost-effective way. Future personalised treatment could further involve personalised feedback using a clinical app.

The project will improve our understanding of the feasibility and acceptability of long-term RMT data collection in adults with ADHD. Feedback from participants and thorough follow-up procedures will help identify challenges for long-term remote monitoring specifically for individuals with ADHD and inform on how to overcome barriers for future implementation of RMT procedures.

## Data Availability

Not applicable – this manuscript does not contain any data.

## Abbreviations

ACLs: Access Control Lists
ADDISS: Attention Deficit Disorder Information Services
ADHD: Attention deficit hyperactivity disorder
AE: Adverse event
ARI-s: Affective reactivity index questionnaire
aRMT: Active app
ART: ADHD Remote Technology
ART-CARMA: ADHD Remote Technology study of cardiometabolic risk factors and medication adherence
AQ-10: Autism Spectrum Quotient questionnaire
AUDIT: Alcohol use disorders identification test questionnaire
AWS: Amazon Web Services
BAARS-IV: Barkley adult ADHD rating scale
BVP: Blood volume pulse
CEIm: Ethics committee of research with medicines
CPT: Continuous performance test
CVD: Cardiovascular disease
DIVA: Diagnostic interview for ADHD in adults
EASO: European Association for the Study of Obesity
EDA: Electrodermal activity
EKS: Elastic Kubernetes System
GAD-7: 7-item generalized anxiety disorder questionnaire
GDPR: General data protection rules
GNG: Go/NoGo
GPS: Global positioning system
HR: Heart rate
IQ: Intelligence quotient
LTE: 13-item list of threatening experiences questionnaire
MINI: Mini international neuropsychiatric interview
pRMT: Passive app
PHQ-8: 8-item patient health questionnaire
PR: Pulse rate
PRV: Pulse rate variability
RADAR-MDD: Remote assessment of disease and relapse in major depressive disorder
RCTs: Randomised control trials
REC: Research ethics committee
REDCap: Research electronic data capture
RMT: Remote measurement technology
RPQ-A: Reactive-proactive aggression questionnaire
RSA: Rivest–Shamir–Adleman
SMEs: Small and medium-sized enterprises
SpO2: Oxygen saturation
SSH: Secure Shell
TIMESPAN: Management of chronic cardiometabolic disease and treatment discontinuity in adult ADHD patients.
